# Emerging Tools to Capture Self-Reported Acute and Chronic Pain Outcome in Children and Adolescents: A Literature Review

**DOI:** 10.3390/medsci10010006

**Published:** 2022-01-25

**Authors:** Alexandra Turnbull, Dean Sculley, Derek Santos, Mohammed Maarj, Lachlan Chapple, Xavier Gironès, Antoni Fellas, Andrea Coda

**Affiliations:** 1 School of Health Sciences, College of Health, Medicine and Wellbeing, The University of Newcastle, Ourimbah 2258, Australia; alexandra.turnbull@uon.edu.au (A.T.); dsantos@qmu.ac.uk (D.S.); muhammad.maarj@uon.edu.au (M.M.); lachlan.chapple@uon.edu.au (L.C.); Antoni.Fellas@uon.edu.au (A.F.); 2 School of Biomedical Sciences and Pharmacy, College of Health, Medicine and Wellbeing, The University of Newcastle, Ourimbah 2258, Australia; Dean.Sculley@newcastle.edu.au; 3 School of Health Sciences, Queen Margaret University, Edinburgh EH21 6UU, UK; 4 Facultat de Ciències de la Salut de Manresa, Universitat de Vic-Universitat Central de Catalunya, 08242 Manresa, Spain; xgirones@umanresa.cat; 5 Priority Research Centre Health Behaviour, Hunter Medical Research Institute, Newcastle 2305, Australia

**Keywords:** digital health, eHealth, mHealth, pain, symptoms, progression, children, adolescent, app, VAS, eVAS

## Abstract

The advancement of digital health provides strategic and cost-effective opportunities for the progression of health care in children and adolescents. It is important for clinicians to be aware of the potential of emerging pain outcome measures and employ evidence-based tools capable of reliably tracking acute and chronic pain over time. The main emerging pain outcome measures for children and adolescents were examined. Overall, seven main texts and their corresponding digital health technologies were included in this study. The main findings indicated that the use of emerging digital health is able to reduce recall bias and can improve the real time paediatric data capture of acute and chronic symptoms. This literature review highlights new developments in pain management in children and adolescents and emphasizes the need for further research to be conducted on the use of emerging technologies in pain management. This may include larger scale, multicentre studies to further assess validity and reliability of these tools across various demographics. The privacy and security of mHealth data must also be carefully evaluated when choosing health applications that can be introduced into daily clinical settings.

## 1. Introduction

Easily accessible digital health solutions may provide strategic and cost-effective opportunities to acquire useful clinical data, even remotely, that could support health-care management in children and adolescents. Clinicians should be supported in accessing emerging-pain outcome measures and employ evidence-based tools capable of reliably tracking acute and chronic paediatric pain over time.

Pain is a complex and multifactorial phenomenon which can negatively impact a child’s health-related quality of life [1]. Pain outcome measures are commonly used to assess the severity of symptoms in children and adolescents [2]. Traditionally, symptom progression has been recorded using either the Wong Baker scale, Numeric Rating Scale, Verbal Rating Scale and Faces Pain Scale-Revised. These tools have been extensively validated and adopted in clinical settings to assess self-reported pain levels [3,4,5,6]. Symptom progression in children can often be misreported, due to the risk of under/overestimation by parents, carers or practitioners [7,8]. Alarmingly, the likelihood of a child receiving pharmacological pain management interventions increases if their pain level is incorrectly recorded [9]. Allied Health Professionals (AHP) require effective and unbiased self-reported pain management tools to support clinical pain management strategies [2].

Clinicians should be made aware of the limitations of the more traditional self-reported paper pain outcome measures that are still commonly used in different paediatric hospital settings [10]. These limitations are mostly based on self-reported paper pain scales being cumbersome, complex to use and possibly at risk of practitioner-interpretation error [11,12]. Despite these limitations, in their now dated trial, Stone et al. (2003) described the use of self-reported paper pain diaries as a valid method to regularly track symptoms, and at different times of the day with the compliance of diaries estimated to be very high (up to 90%) [13]. Compliance of self-reported paper pain diaries is much lower than originally estimated and increases the chance of participants backfilling data [13]. This limitation increases the risk of recall bias, reduces the accuracy of pain diaries and highlights the risk of incorrect and inconsistent use of certain self-reported pain tools.

Recently, modern technologies have emerged which utilise novel self-reported pain outcome tools, such as eOuch, SUPER-KIDZ and iPadVAS. Advances in technology through electronic self-reported pain recording has the potential to address the existing limitations of self-reported paper pain outcome measures by providing the opportunity to complete intermittently throughout the course of the day with constant access to electronic devices [12]. Pain and distress outcomes in children have been extensively assessed for methodological quality [14,15]; however, further research is required in this paediatric field to facilitate the introduction of new smart technologies into daily clinical practice. This could potentially increase compliance and accuracy in recording self-reported pain levels in children and adolescents. This literature review will discuss the advantages and limitations of emerging self-reported pain outcome tools and compare them to traditional pain outcome measures.

## 2. Materials and Methods

A search was conducted using both Medline, PubMed, Pedro and Psycho information from January 1990 to October 2021, to review current literature and to collate articles, which were assessed in terms of inclusion/exclusion criteria and quality of evidence.

The search was conducted using the following keywords: Pain outcome measure, pain assessment, pain measurement, children, adolescent, electronic, smartphone, smart device, smart-technology, validation, feasibility (Table 1). Inclusion/exclusion criteria for this literature review are presented in Table 2.

Two reviewers (A.T., A.F. and A.C.) independently screened the titles and abstracts of all studies identified by the search. Full-text articles of potentially eligible studies were retrieved by A.T. and A.F. and independently screened by A.F. and A.C. Authorship and results were not masked. Disagreements between the two authors regarding full-text inclusion were resolved by a third reviewer (D.S.). If disagreements were not resolved successfully by the third reviewer, study authors were to be contacted, although this was never required. A.T. and A.F. extracted data from the included studies using a standardized pilot-tested form, and a second author (A.C.) checked all extracted data. If there was any absent or uncertain information, study authors were contacted. Inconsistencies in data extraction were discussed between A.T., A.F. and A.C. and, if needed, through arbitration by D.S.

## 3. Results

**Figure 1:**
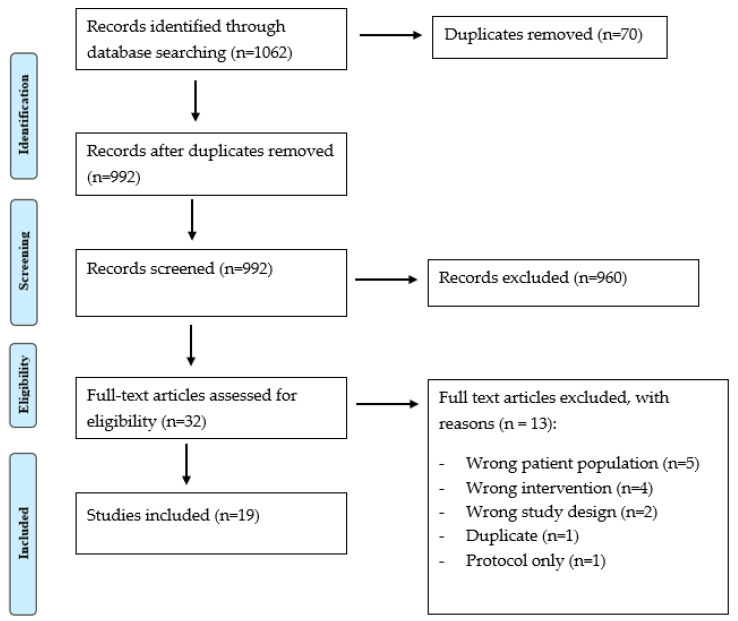
PRIMA flow chart.

Overall, 1062 papers were obtained from the conducted search and contributed to this literature review, of which 70 duplicates were found and removed. Overall, 992 titles and abstracts were screened, and 960 were excluded due to lack of relevance. A total of 19 articles met the criteria for inclusion in this study [16,17,18,19,20,21,22,23,24,25,26,27,28,29,30,31,32,33]. The main reason for rejecting papers was the age range inclusive of children (0–18), without being exclusive to this age range. Figure 1 presents the PRISMA flow diagram. A summary of the findings and limitations of emerging pain outcome measures is available in Table 3.

## 4. Discussion

Based on the obtained results, this discussion section outlines two distinct systems used to record acute and chronic pain progression:

(A) Electronic Devices, such as PC or laptops, where data is stored on the internal memory and then instantaneously uploaded to the cloud when a network is available or moved to an external device.
(B) Smart Devices capable of WiFi connectivity or linked to 3G or 4G mobile networks. These devices are typically cordless, highly portable, with the option to capture the location of service, enable interactive text and voice call recording with the ability to take photos and videos. For example, these may include, but are not limited to: a Smartphone (i.e.: iPhone), tablet (i.e.: iPAD, Samsung Galaxy) or smart watch (i.e.: Apple watch). The advantages and disadvantages of these emerging technologies tested in children and adolescents, will form the basis of the following discussion.

### 4.1. Electronic Devices Used to Capture Symptom and Pain Progression

#### 4.1.1. Electronic Diaries

The use of electronic pain diaries has the potential to increase the compliance of recording data and may reduce recall bias compared to paper pain diaries (16). Palermo et al. (2004) investigated 66 children aged 8–16 with Juvenile Idiopathic Arthritis (JIA) or headaches. The paper diary group had significantly more omissions and errors compared to electronic pain diaries (which had none). Additionally, the electronic diaries had significantly more days completed over a week compared to paper pain diaries. Stinson et al. (2004) assessed a multidimensional electronic pain diary (eOuch) in terms of its real-time construct validity and feasibility. The study was conducted in an age range of 9–18. The methodological approach accounted for the fluctuations of active arthritis symptoms that unfortunately occur throughout the day in JIA; therefore, the patients were asked to record their pain level at different times (i.e.: morning, lunch-time, evening). This addressed a limitation of prior research by Palermo et al. (2004) [16], in which they only asked participants to measure symptoms at the end of the day.

#### 4.1.2. PDA

Wood et al. (2011) concluded that there was a reduced recall bias through the introduction of an electronic PDA with the faces pain scale compared to paper [22]. The study recruited 202 children, 4–12 years of age. Interestingly, results indicated that they found similar pain scores on both measures, and the mean absolute discrepancy was not statistically different.

#### 4.1.3. Web-Based Multidimensional Pain Measure

Luca et al. (2017) assessed a web-based multidimensional pain measure for children and youth with JIA [23]. This included 71 participants aged 8–18 and 29 parent–child dyads aged 4–7. Findings from this study concluded that SUPER-KIDZ has good internal consistency, responsiveness and satisfactory test–retest reliability [23]. All of these tools present various advances compared to traditional paper based outcomes.

#### 4.1.4. Pain Measurement Tools Comparison

There are several limitations identified in these studies that need to be included. Firstly, Palermo et al. (2004) did not address changes in pain throughout the day. Stinson et al. (2008) had high compliance; however, 22% of the data was missing, which may lead to a biased estimate of average weekly electronic pain diaries. Wood et al. (2011) included a cohort of chronically ill and healthy children attending day surgery within the hospital outpatient setting. It could be argued that the non-homogeneity of the observed groups could have significantly impacted the overall results, especially when dealing with chronically ill paediatric patients with complex medical and pharmacological history. The time between administration of the paper FPS-R and PDA FPS -R was limited to any time frame that was less than 30 min. As a consequence, multiple results were excluded from the study due to the time between measures being less than 1 min. It is possible that the lack of standardisation of the time frame between administration of self-reported tools impacted the overall findings. Nurses were asked to extensively explain how to use the PDA version of the scale, and it is possible that there was preferential bias associated with the PDA faces pain scale. Alarmingly, due to different French hospital legislations, no ethics approval was obtained in order to conduct this paediatric research.

Finally, in comparison to paper-based traditional outcome measures, the electronic pain diaries clearly showed the following: reduced recall bias, the ability to measure pain at different times of the day, and a reduction in the amount of errors in pain diaries compared to paper based pain diaries. Web-based tools were able to present an array of outcome measures within the web-based program, which are validated across a relatively large age range in children (ages 4–18).

### 4.2. Smart Technology to Monitor Symptom and Pain Progression

Sanchez-Rodriguez et al. (2015) assessed the validity and agreement of intensity reports of an application (the Painometer) that had four different pain intensity scales, against their traditional counterparts. Overall, 180 participants were recruited from ages 12–19. The results from this study showed that the scales were highly interchangeable, with a confidence interval of 80%. Stinson et al. (2015) assessed the reliability of a multidimensional smart phone app in children and adolescents with cancer [19]. The study consisted of two components to determine validity, reliability and responsiveness. Authors concluded that real-time data collection had the potential to reduce recall bias and improve the understanding of associated symptoms in paediatric cancer patients. Sun et al. (2015) investigated the agreement between their application, called ‘Panda’ and original paper/plastic versions of the FPS-R and CAS to determine children’s preference between each of the scales [27]. Sun et al. (2015) reported a significant preference (*p* < 0.005) for their application compared to the use of their traditional paper counterparts. This study involved 62 participants and provided insightful evidence into a younger cohort of participants and the use of smart technologies to track pain progression.

There are multiple limitations to these studies that should be considered. Sanchez-Rodriguez et al. (2015) included multiple outcome measures; however, they did not randomise the presentation order of the scales. This has been previously shown to not influence the obtained rating [3]; however, it may influence results especially when gathering data with children. The authors also describe the time (30 min) between two assessments as not being adequate to prevent memory effects. Additionally, due to the sample population, participants were asked to recall maximum pain intensity in the last three months. This may present some issues in terms of reliability measures. Stinson et al. (2015) described a limitation in capturing symptom progression at certain times of the day (morning and night) compared to capturing symptom progression on an individual basis (i.e., when a participant exhibits pain). This may have led to omitting fluctuations in pain during the day and may have led to potential bias. Furthermore, study two, which assessed responsiveness and feasibility, was unable to successfully recruit the number of the required participants. Sun et al. (2015) failed to assess the reliability of their application. Results found a systematic bias towards the smartphone application specifically with regard to the limits of agreement for clinical significance, the cause for this was not discussed. Additionally, 27% of all pain assessments of FPS-R were of a value of 0. This indicates that further research is needed to assess a wider range of pain intensities using this application, due to a possible end-point bias. Additionally, as reliability was not determined, these values may not have been entirely accurate.

Turnbull et al. (2020) and Martinez Garcia et al. (2020) used InteractiveClinics App and the PainApple App, respectively to monitor symptoms using validated technologies. Results showed moderate to good ICC amongst healthy children and adolescents when interchanging the eVAS compared to the traditional pVAS. Instead, Martinez et al. (2020) measured pain and other post-operative outcomes at 30 min intervals. The PainAPPle appeared to be a valid instrument to assess the management of acute pain in pediatric patients.

### 4.3. Electronic and Smart Technology Compared to Traditional Outcome Measures

#### 4.3.1. Clinical Implications and Considerations

The research evidence regarding the use of emerging outcome measures is broad and provides promising developments for clinicians to accurately measure and monitor pain and symptom progression (Table 2). Modern technologies have the potential to capture real time data and reduce recall bias, aiming to improve the reliability of clinically tracking symptom progression in children and adolescents. Clinicians should be made aware that most of the studies conducted so far were not multicentered and ranged across various paediatric demographics (hospitalised children, or healthy children). Thus, it is difficult to establish the transferability of these results across various paediatric clinical settings. Additionally, there is limited evidence to demonstrate the feasibility, usability and responsiveness of these applications. Whilst most studies asked a child to indicate a preference for each device [26,27,34], speech recording devices and large scale feasibility trials have not yet been performed on the use of smart technologies in monitoring pain.

The results of this review also highlight the considerable effort invested in developing smart technologies in the field of paediatric rheumatology. These promising advancements, specifically in JIA research, demonstrated how the eOuch (Stinson et al., 2014) and the JIApp (Cai et al., 2017), provide validated innovation, able to acquire pain assessment data that may be used to effectively informed clinical decision making and raise patient’s awareness of their condition.

#### 4.3.2. Data Privacy

A prevalent issue involving the use of smartphone applications is the privacy and security of data collected through these platforms. Often, users of smartphone applications are unaware of how their data is used and managed [35]. Recent evidence suggests that there are concerns over the lack of privacy policies included in smartphone applications used for certain diseases such as diabetes and dementia [36,37]. Considering the sensitive nature of chronic pain conditions, data privacy and security should be taken into account when making the transition into emerging tools to monitor symptom progression [38].

### 4.4. Digital Health and Clinical Limitations

Considerations that should be taken into account in the future is the issue that smart technologies may not be easily affordable by all children and their parents/carers. The financial disadvantage is identified as a major hurdle in electronic devices to measure symptom progression [22]. The use of computers, laptops, PDAs and electronic pain diaries does represent an extra financial burden for the families and may exclude a child or adolescent from accessing the most suitable technology to track pain. Nevertheless, positive evidence highlights that 67% of primary school aged children, and surprisingly 36% of preschool aged children already own a mobile based screen device [39]. By supporting mobile based applications that can be downloadable directly onto an existing smartphone, it may present with a significant saving solution for families.

Excessive screen time presents a possible health issue for children and adolescents [38]. A 2020 systematic review reported a concerning trend of correlation between time spent by children using screens and developing of myopia [40]. Whilst the effects of excessive screen usage is unclear [40], other authors still recommend regular parental supervision in order to minimise these potential side effects [38].

Future research should focus on addressing the limitations outlined in Section 4.4. Detailed usability and acceptability trials should be conducted to test these smart technologies prior to their introduction to young symptomatic patients. Larger powered clinical trial and systematic reviews are urgently required to validate the different emerging digital health technologies to support acute and chronic pain management in children and adolescent. A cost-effectiveness evaluation may also need to be considered to determine if digital health is capable of reducing the overall cost to the health care system. Finally, researchers and the industry need to work closely together to promote the highest standards of data privacy, whilst using country-based secure servers to store sensitive data.

## 5. Conclusions

Emerging technologies may have the potential to improve the methods by which Allied Health Professionals monitor symptom progression amongst children and adolescents with the capacity to improve the responsiveness in their clinical management. Growing evidence indicates how the recently developed mHealth Apps, designed to record pain intensity scales, are highly interchangeable compared to their traditional paper versions. More research is required to further investigate new reliable tools capable of recording pain. In particular, larger, multicentre randomised controlled trials are needed to consolidate the use of smart technologies in pain management in children and adolescents. The feasibility and responsiveness of these mHealth tools need to be carefully studied with particular focus on the possible financial impact and savings for the health care system. In conclusion, it is of paramount importance that the privacy and security of mHealth data should be carefully considered when choosing health applications that can be introduced into daily clinical settings.

## Figures and Tables

**Table 1:** Search Strategy.

1	Pain outcome measure
2	Pain assess *
3	Pain measurem *
4	1 or 2 or 3
5	Child *
6	Adolescent.tw
7	5 or 6
8	Electronic *
9	Smartphone
10	Smart device
11	Smart-technolog *
12	8 or 9 or 10 or 11
13	Validation
14	Feasibility
15	13 or 14
16	4 and 7 and 12 and 15

**Table 2:** Inclusion exclusion criteria.

Inclusion Criteria:	Exclusion Criteria:
	Articles that do not explicitly state the exclusion of participants that have:
English language	Neurological disorders such as sensory processing disorders, or intellectual disabilities that impede the participants’ perception of pain.
Ages 0–18	A physical disability that impairs the ability to carry out self-reported pain scales i.e., visual impairment.
Peer reviewed articles	Editorials will not be included
Conference abstracts) are eligible only if we can identify the full-text report.	Study protocols for future or ongoing evaluations to capture self-reported acute and chronic pain outcome in children and adolescents based interventions.

**Table 3:** Summary of findings and limitations of emerging pain outcome measures included.

Name	Study	Methodology and Sample	Findings	Limitations
Electronic pain outcome measures				
Electronic pain diary	Palermo et al., 2004	Randomized clinical trial. n = 60, age range 8–16 mean=12.3 Headaches or Juvenile Idiopathic Arthritis	Children with e-diaries completed more days compared to p-diaries. P-diaries more errors.	Only included pain tracking at the end of the day, not multiple times of the day
Computer Face Scale	Fanciullo et al., 2007	Cohort observational study evaluating feasibility of the computerised version of the Wong Baker Face scale. 54 in-patient children with mean age of 10.7; and a second convenience sample of 30 childrens with mean age of 7.2. Each sample used to test two objectives.	Authors reported that the majority (76%) of participants preferred to use the computer version over paper. Moreover, authors showed that children were able to show varying levels of emotion when expressing pain levels.	Data only collected at one time point and timeframe was not explicit. No control group. Difficult to quantitatively compare with other scales that use 0–10 numerical values.
eOuch	Stinson et al., 2008	Descriptive study design. 13 adolescents with Juvenile Idiopathic Arthritis (JIA).	Participants required to complete eOuch 3 times per day. Most participants reported the ediary was easy to use. Phase 1 of study had 73% compliance and phase 2 had 70%.	Small sample size and sample only from one tertiary pediatric centre. Same patients were used in both usability and acceptability studies.
eOuch	Stinson et al., 2008	A descriptive study design. Study 1: n = 76, age range 9–17, mean 13.4 Study 2: n = 36, age range 8–17, mean = 12.6 Rheumatology clinics	Data was collected by the children. Evidence of construct validity and feasibility of eOuch pain diary in adolescents with JIA. Provided more information (3 times a day) compared to Palermo et al.	22% of data was missing potentially leading to a biased estimate of average weekly electronic pain ratings.
personal data assistants (PDA) FPS	Wood et al., 2011	Observational, multicenter, randomized, cross-over, controlled, open trial. n = 202, age range 4–12.	Data was collected by hospitalised children. Mean levels of pain scores were 3.1 ± 2.3 and 3.2 ± 2.3 for paper and PDA scores, respectively. The mean absolute discrepancy between the two versions was not statistically different significant from zero	Participants from multiple wards—chronic disease and also day surgery. None of the studies had a mean time between the assessments of “less than 30 min”
Computer Face Scale	Cravero et al., 2013	Validation study of the Computer Face Scale. Included 40 children aged 5–13 who underwent a tonsillectomy at Children’s Hospital at Dartmouth-Hitchcock Medical Center. Participants used a Dell Mobile PDA to display the face scale and arrows were used to cycle between each expression.	When comparing CFS to the verbal rating scale and wong baker scale, authors concluded good validity scores: “The correlation between the pain ratings from the Computer Face Scale and the Wong-Baker Faces Scale after surgery was 0.83”.	Sample population was limited to those undergoing surgery and therefore we were unable to generalise to other populations. No mention of CFS available for use on mobile phones and/or app stores across multiple devices.
eOuch	Stinson et al., 2014	Construct validity study in children with JIA. Comparing momentary and recalled pain measurements with eOuch. 70 adolescent JIA participants.	Between-person momentary and recalled pain measurements showed a moderate Interclass Correlation Coeficient (ICC). Within-person measurements displayed weak ICC.	Sample sourced from one clinic. Study did not include a practice session. Weekly momentary analysis may have been influenced by 22% missing data reported.
SUPER-KIDZ	Luca et al., 2017	Clinimetric study using prospectively collected repeated measures Study 1: n = 71, age range 8–18 Study 2: n = 29 (parent child dyads), age range 4–7	For study 1, data was collected by the children Good internal consistency, responsiveness and satisfactory test–retest reliability.	Small sample size Reliability study compromised due to unstable pain levels
BAPQ-C	Jordan et al., 2020	Fourteen adolescents with chronic pain (13 females; 13–16 years) were recruited from a hospital-based residential pain management programme. Qualitative study focusing on exploring the feasbility of the electronic version of Bath Adolescent Pain Questionnaire.	Authors reported high acceptability of the BAPQ-C. 93% of participants reported that the BAPQ-C was both ‘quicker’ and ‘easier’ to complete than the BAPQ. Only one participant preferred the paper version.	Small and specific sample population. Further validation required in patients outside hospital residential care.
**Smart Technology Pain Outcome Measures**				
Pain Squad	Stinson et al., 2013	Usability, feasibility, compliance, and satisfaction study. Qualitative interviews followed by compliance and satisfaction data were obtained. 15 adolescents with cancer with average age of 13.	Participants during interviews provided feedback on the app and authors made adjustments accordingly. “88% of questions were rated as “important” or “very important by the majority (> 50%) of adolescents”. Compliance was high with mean of 81%. Authors also reported high satisfaction among the sample.	No direct examination of high compliance rates. App only accessed with iPhone or other apple devices. Information from interviewed participants was not verified with a follow-up interview.
Painometer	Sanchez Rodriguez et al., 2015	Cross-Sectional Observational Study n = 180, age range 12–19 mean = 14.8 4 updated traditional outcome measures updated to smart devices	Data was collected by the children. 80% confidence interval—determined that they were interchangeable	Asked participants to remember maximum pain over last 3 months. Presentation order of scales was not randomised
Pain Squad	Stinson et al., 2015	A prospective descriptive study design with repeated measures was used to test the construct validity, reliability, and feasibility. Study 1: n = 92, age range 8–18, mean 13.1. Study 2: n = 14, age range 9–18, mean 14.8 Pediatric cancer patients	Data was collected by the children Found that the multidimensional app was valid, reliable and feasible within a pediatric cancer setting	Small sample size in study 2—not enough participants recruited Measures collected morning and evening—may have missed other fluctuations leading to bias
Smartphone FPS-R and CAS	Sun et al., 2015	observational, randomized, cross-over-controlled, open trial. Study 1: n = 62, age range 4–12, mean 7.5 Study 2: n = 66, age range 5–18 mean 13 Children scheduled for surgery with anticipated post operative pain	Data was collected by the children Panda correlated strongly with original scores. Mean pain scores higher in application compared to original tool—systematic bias, within clinical significance (80%)	Not multi dimensionsional—27% of scores were 0 on the FPS-R. Did not assess reliability of application. One sample location.
JIApp	Cai et al., 2017	Design, develop, and evaluate the acceptability and usability of JIApp. 3 phase study on children with JIA. Participants ranged from ages 10–24 across the phased study. Three themes: (1) Remote monitoring; (2) Treatment adherence; (3) Education and Support.	Ability for patients with JIA to report and monitor several parameters associated with their disease including but not limited to pain, joint symptoms, psychological well-being, activity limitation. Young JIA patients reported a mean acceptability rating of 4.29 and expressed multiple benefits of the app.	Limited sample size. Will require further validation in a larger clinical trial.
Pain Squad+	Jibb et al., 2017	An cohort prospective design of adolescents ranging from 12–18 who were currently undergoing cancer treatment. 40 participants were recruited with a mean age of 14.2.	Overall adherence of Pain Squad+ was 77.2%. Acceptability e-scaled showed a minimum average of 3 in all items assessed indicating satisfactory acceptability.	Single group design. No control group. Pilot study and therefore requires further investigation on a larger sample size.
iCanCope PostOp app	Birnie et al., 2019	User-centered design study with 2 principle phases. (1) Semi-structured interviews, (2) 2-stage Delphi Survey. 19 children with mean age of 15.26 who underwent surgery within a 7-day period were recruited. iCanCope: a smartphone-based app for children and adolescents’ self-management of acute postoperative pain.	All participants reported the three proposed features of the app as important (pain tracking, pain advice, and goal setting). Multiple features were proposed by participants, parents and health care workers. These include but are not limited to: Pain advice within the app; goal setting; direct communication with health care providers and medication tracking were also proposed.	Convenience sample. Potentially limited by a lack of comprehensiveness of all types of surgeries for potential end users.
Interactive Clinics App	Turnbull et al., 2020	Cross-Sectional Observational Study. 47 children and adolescents (mean age 13.9 years, SD 2.89 years; range 10–18 years).	Authors concluded moderate to good ICC when interchanging the eVAS and pVAS.	Convenience sample. Possibly lower reliability in children/adolescent sample due to differences in scale sizes fo VAS measuring line.
PainAPPle^®^	Martínez García et al., 2020	Descriptive cohort study of 44 paediatric patients post surgery. Mean age = 11.3.	Data were collected by children after they recovered from their anaesthetic post-surgery. PainAPPle was used at 30 min intervals to measure pain and other post-operative outcomes. Statistically significant correlations were produced when comparing the electronic and paper versions of PainAPPle.	Specific population sample. Not generalisable. Requires further testing for validation.
iCanCope app	Lalloo et al., 2021	Feasibility and pilot RCT for the iCanCope app in adolescents with JIA. 60 adolescents with JIA recruited and randomised to a control or trial intervention. Mean age = 15.0. Trial invervention/condition was the iCanCope app + self management features. The control group only received the iCanCope app (no self-management).	Both study conditions were deployed with high success. Pain intensity improved in both groups by 1.73 (intervention) and 1.09 (control). No significant changes in quality of life or pain-related activity limitations. Overall, the app was adhered to well and acceptable to most adolescent JIA patients with pain.	Requires a third arm (with just usual care, i.e., no app) to assess the effectiveness of iCanCope on outcomes in children with JIA.

## Data Availability

Data available on request due to ethical restrictions. The data presented in this study are available on request from the corresponding author. The data on this review are not publicly available due to ethical reasons.
